# *"The solution needs to be complex." *Obese adults' attitudes about the effectiveness of individual and population based interventions for obesity

**DOI:** 10.1186/1471-2458-10-420

**Published:** 2010-07-15

**Authors:** Samantha L Thomas, Sophie Lewis, Jim Hyde, David Castle, Paul Komesaroff

**Affiliations:** 1 Consumer Health Research Group (CHaRGe), Primary Care Research Unit, Monash University, Melbourne, Australia; 2 Department of Health, Victoria and Faculty of Medicine, Deakin University, Melbourne, Australia; 3 Department of Psychiatry, University of Melbourne, Melbourne, Australia; 4 Department of Medicine, Monash University, Melbourne, Australia

## Abstract

**Background:**

Previous studies of public perceptions of obesity interventions have been quantitative and based on general population surveys. This study aims to explore the opinions and attitudes of obese individuals towards population and individual interventions for obesity in Australia.

**Methods:**

Qualitative methods using in-depth semi-structured telephone interviews with a community sample of obese adults (Body Mass Index ≥30). Theoretical, purposive and strategic recruitment techniques were used to ensure a broad sample of obese individuals with different types of experiences with their obesity. Participants were asked about their attitudes towards three population based interventions (regulation, media campaigns, and public health initiatives) and three individual interventions (tailored fitness programs, commercial dieting, and gastric banding surgery), and the effectiveness of these interventions.

**Results:**

One hundred and forty two individuals (19-75 years) were interviewed. Participants strongly supported non-commercial interventions that were focused on encouraging individuals to make healthy lifestyle changes (regulation, physical activity programs, and public health initiatives). There was less support for interventions perceived to be invasive or high risk (gastric band surgery), stigmatising (media campaigns), or commercially motivated and promoting weight loss techniques (commercial diets and gastric banding surgery).

**Conclusion:**

Obese adults support non-commercial, non-stigmatising interventions which are designed to improve lifestyles, rather than promote weight loss.

## Background

Obesity is a national health focus in Australia. In 2007/8 the Australian National Health Survey, based on hip and waist circumference, Body Mass Index, and self report data, concluded that 25% of individuals over the age of 18 were obese, 37% overweight, 37% normal weight and 2% underweight. More adult males (68%) were overweight or obese than adult females (55%)[1]. A range of solutions have emerged in Australia to counter the increasing rates of obesity. At the individual level, a plethora of commercial solutions - from *The Biggest Loser *to bariatric surgery - have emerged for those wanting to reduce their weight and improve their health and wellbeing. At the population level, a number of inquiries and taskforces have recommended a variety of responses, including regulation, increased social marketing campaigns and environmental changes to encourage increased physical activity[2,3].

At present there is only limited evidence to support interventions that lead to long term sustained change in health and behaviour regarding obesity. Individual level evidence shows that whilst commercial dieting solutions may be successful for short term weight loss they may also lead to long term weight gain[4]. Individually tailored physical activity programs have shown more promise, but again lack longitudinal evidence showing long term behaviour change[5]. Similar limitations pertain to models which advocate shifting the emphasis from weight loss in overweight adults to more holistic models of health and wellbeing[6,7]. These non-dieting approaches have developed out of an extensive literature demonstrating the damaging health impacts of dieting behaviour for obese individuals, including disordered eating behaviour,[8] long term weight gain,[4] and the negative health consequences of weight cycling[9]. They show that whilst weight loss may be more modest, individuals have improved self esteem, sense of self control and self efficacy, and are more likely to engage long term in physical activity[10].

At the population level, large-scale anti-obesity social marketing campaigns may increase an awareness of the need to 'eat less and move more', but may do little to effect behaviour change[11]. Furthermore, these types of interventions may inadvertently increase social stereotypes and stigma by focusing on the simplistic outcomes of risk behaviours (obesity) rather than the causes of these behaviours (environmental factors, junk food marketing and over consumption)[12]. Public health campaigns and initiatives, such as menu labelling [13], the regulation of junk food marketing [14] and encouraging physical activity [15] - particularly in children - provide new opportunities for education and lifestyle change. Yet few population based responses offer individuals a 'road map' of supportive solutions that take into account the well demonstrated complexities of obesity[16]. As such, many individuals find themselves treading the well worn path of commercial dieting as a solution to 'being fat'. In sum, these findings may suggest a two pronged strategy is needed in our efforts to address obesity: 1) The need for primary prevention (population based) strategies to prevent overweight and obesity - particularly in children; and 2) Secondary prevention strategies to help facilitate healthy, non-dieting approaches for those individuals who are already overweight or obese.

In this environment, research has investigated the extent to which the general public supports different types of community interventions for obesity - particularly in children[17]. These studies show widespread support for a range of different types of strategies at the individual level - including behavioural, lifestyle and 'no dieting' interventions. At the population level, there is growing support for education campaigns, and regulation (such as the banning of junk food advertising to children)[18]. What is less clear is an understanding of what obese individuals think would be beneficial in addressing the obesity epidemic both at the individual (care) and population (prevention) levels. Consumer involvement, perceptions and engagement are well recognised as cornerstones for developing effective interventions within communities and in improving the translated outcomes of intervention programs. Yet, there has been very limited research seeking to understand the perspectives, attitudes and opinions of obese adults about current approaches to obesity[19]. Research with other health groups (e.g. mental health consumers, women with breast cancer, and people living with HIV/AIDS) has shown that lay knowledge and lived experiences provide valuable information to guide policy, interventions and service delivery[20,21]. In Australia and in other countries, numerous policy initiatives have called for public involvement in health and social research, *"at every stage of the research where appropriate*"[22,23]. However, in obesity, the voices of obese adults remain deafeningly silent.

This paper presents qualitative research exploring obese individuals' attitudes and opinions towards population (regulation, media campaigns and public health initiatives) and individual (specialised fitness programs, gastric banding surgery and commercial dieting) interventions for obesity in Australia.

## Methods

### • Approach

In-depth qualitative interviews with a community sample of obese Australian adults. The data presented in this paper were part of a broader Australian study, *"Obesity: Have Your Say!"*, which explored the experiences, attitudes and opinions of individuals with a Body Mass Index ≥ 30. Other papers from this study have detailed the risk perceptions of participants according to the severity of their obesity [24] and mental health, coping strategies and body image [25]. Ethical approval was gained from the Monash University research ethics committee.

### • Recruitment strategy

Our recruitment strategy was guided by illness experience literature which shows that individuals' experiences may vary according to demographic, geographic and socio-cultural factors, and also the severity of their illness. We employed a combination of theoretical, purposive, and strategic sampling methods[26,27]. For example, strategic sampling methods ensured that we included individuals with a wide variety of attitudes to weight, weight loss and other related issues; those who were attempting to lose weight; those who were attempting to live a healthy lifestyle; those who had abandoned all efforts to address their weight; and those who were happy with their weight. We recruited individuals using media publicity, on-line advertisements, recruitment via health professionals, personal trainers, commercial and community-based weight loss centres, as well as community advertisements. It is important to note that the study did not seek to represent the experiences of different ethnic groups. This is because a study of this type and size would not have been able to adequately represent the experiences and perspectives of culturally and linguistically diverse groups in Australia.

### • Data Collection

Telephone interviews were conducted between April 2008 and March 2009 and were between 60 and 90 minutes in duration. Interviews were digitally recorded and transcribed within seven days of the interview by a professional transcribing company.

In this part of the broader study, participants were presented with prompt examples of six different types of obesity interventions currently used in Australia. We chose contemporary examples that we anticipated would be commonly recognised by participants.

1) Media based social marketing campaigns: The Cancer Council's *Reduce-your-waist *campaign which linked increased waist circumference with increased risk of cancers.

2) Public health interventions and initiatives: The Victorian Government's *Go for Your Life *campaign which aimed to promote healthy lifestyles; and the Australian Federal Government's *Go for 2 and 5 *campaign, which aimed to promote fruit and vegetable consumption.

3) Regulation: Banning junk food advertising aimed at children

4) Obesity surgery: Public funding for gastric banding surgery - the most commonly used obesity surgery in Australia.

5) Commercial diets: *Weight Watchers *and *Jenny Craig.*

6) Specialised fitness programs: Female only gyms (such as *Curves*) and public funding for personal trainers.

Participants were asked to discuss each intervention and whether they thought it was effective in improving health and wellbeing for obese individuals. We used probe questions to stimulate discussion when required (i.e. *Why do you think that? Could you explain that some more?*).

### • Data Analysis

We used a descriptive thematic approach to the analysis of the data. Data were analysed by ST and SL, using a constant comparative method of analysis[28,29]. This involved reading and rereading the transcripts, coding and identifying themes. We interpreted the responses to each of the distinct interventions, looking for themes within groups. We investigated similarities and differences in responses, and the reasons why these occurred. We tested the reliability of ST and SL's interpretations by randomly selecting ten transcripts and comparing the data analysis across these transcripts. The thematic results were presented both to the broader research team for discussion and interpretation, and to a group of obese consumers who discussed and provided feedback on the interpretation of the data.

The research findings are illustrated using quotes from participants. Because of the large number of participants, some of the responses have been quantified allowing us to identify the proportions of individuals who responded in certain ways. Where we have not used numbers we use the term 'a few' to refer to less than a quarter of participants; 'some' to refer to 25-50% of participants; 'many' to refer to 50-75% of participants; and 'most' to refer to over 75% of participants.

## Results

### • General Characteristics

The characteristics of participants are summarised in Table 1. Initially, 172 individuals expressed interest in participating in this study. Eight decided not to participate because of the time involved, or because they thought the study was promoting a new weight loss intervention. Twenty-two were excluded because their BMI was less than 30, or because they did not live in Australia. The average age of participants was 44.8 years (19-75 years). Three-quarters of participants were female (n = 106, 75%). Whilst nearly two-thirds of participants had a tertiary education, they were not necessarily from high socio-economic groupings. Over three quarters of participants had an income before tax of less than $100,000 AUD.

**Table 1:** Participant Demographics

Demographic category	n (142) (%)
*Gender*	

Female	106 (74.6%)
Male	36(25.4%)

*Age*	

Mean	44.8
Range	19-75

*BMI*	

Mean (n = 141)*	39.3
Range	30.0-71.7

*Marital status*	

Single	50(35.2%)
Married/De facto	92(64.8%)

*Education*	

< High school	20(14.1%)
High school graduate < University degree	33(22.2%)
University or postgraduate degree	89(62.7%)

*Income before tax (AUD)*	

<50,000	48(33.8%)
50,000-100,000	59(41.5%)
>100,000	33(23.2%)
Not revealed	2(1.4%)

*One participant did not reveal height or weight to calculate their BMI

### • Population based interventions

#### Regulation

About two thirds of participants thought that regulation was one of the most effective solutions for the obesity epidemic (87, 61%). More women (70, 66%) than men (17, 47%) thought that regulation such as the banning of junk food advertising to children was an effective approach. Women also went on to speak about banning junk food in school canteens. This perhaps reflects women's key roles in the purchasing and provision of food and food choices within the family home. Participants spoke about the commercial interests and marketing tactics of multinational corporations in influencing food choices, and were particularly supportive of regulation to protect children and young people, who were perceived to be most vulnerable, from this marketing:

*"McDonalds and all of those, they run special campaigns to get kids in and to get them more than once a week, like having a series of toys and getting a new one each week...keeps them coming back." *(Female, aged 53)

A few participants thought that taking away the visual stimulation of junk food advertising would positively influence the purchasing behaviours of their family unit:

*"I think that's a fantastic move because I think children are influenced by television advertising. I guess we all are in some ways." *(Female, aged 53)

However, some participants were concerned that regulation, and in the particular taxing of 'unhealthy' foods was disempowering by taking away individuals' personal choice and responsibility. They were also concerned that regulation would reinforce moral judgements about food choices. Men in particular spoke about freedom of choice around food options:

*"It's not the State's responsibility or the health profession's responsibility to be dictating what we can and can't eat, or how we can and can't eat." *(Male, aged 42)

Others stated that regulation of junk food advertising did little to address the complexity of food choices in households. These participants commented that the banning of junk food advertising was overly simplistic because it ignored the fact that food marketing occurred in many different places - not just on television:

*"That's not going to make a difference because it's always out there and kids know when they go to the shops it's all, at the supermarket, at the checkout it's at your face." *(Female, aged 54)

Some participants commented that the move toward regulation was yet another attempt to provide a quick fix to a "*complex*" health issue:

*"It's a complex range of choices that people are faced with and so the solution needs to be complex" *(Male, aged 39)

Whilst participants felt that some types of regulation, particularly in relation to the marketing of junk food to children, may increase people's capacity to make healthy and informed choices about food, they also stressed that any regulation should occur alongside education and support.

#### Public Health Initiatives

Fewer than half (60, 42%) of participants thought that current public health prevention initiatives were effective in engaging individuals in lifestyle changes. However, this figure needs to be treated with some caution as some individuals had not heard of any of the 'prompt' examples we provided in our study. Despite this, participants who had heard of *'Go For Your Life' *said that these types of campaigns had positively impacted on their behaviour in small ways. This was because they focused on education, practical examples and support. Some described how they had applied the examples given in the initiatives to their own lives:

*"I tend to park further away at the supermarket so I have to walk further." *(Female, aged 54)

Participants also trusted interventions from public health agencies as they thought these were more likely to be evidence-based:

*"I respect that what they are saying is not just a marketing ploy but it is a fact." *(Female, aged 49)

Participants liked that these initiatives such as *'Go For Your Life' *encouraged lifestyle changes rather than weight loss. Because of this they were perceived as less stigmatising, and more supportive in helping individuals to make long term lifestyle changes. For example, they felt that '*Go For Your Life' *encouraged people to focus on positive, practical and realistic strategies to address their health and wellbeing, rather than promoting 'quick fix' weight loss:

*"Encouraging something that you can incorporate into your everyday life is better than something that you do for a while and then you give up." *(Female, aged 59)

They also liked the fact that these initiatives encouraged physical activity as an enjoyable, daily activity that families could participate in together:

*"I actually really like it because a lot of the activities are centred around families and getting out there as a family." *(Female, aged 36)

*"I mean encouraging people to be physically active and giving them strategies to do so, like take the stairs instead of the elevator that sounds alright." *(Female, aged 32)

The downside for some people was that they felt that campaigns were predominantly targeted at children and young people. Furthermore, many felt that they provided only limited strategies about how you could get moving if you were already obese. Many said that the public health initiatives were designed around simplistic assumptions about what the 'average person' could achieve and provided limited secondary information for people who may have had more complex health needs, or who may feel stigmatised when taking part in these activities.

#### Media Campaigns

One third (47, 33%) of participants thought that media campaigns were an effective way of addressing the obesity epidemic. However, many felt that current campaigns made inaccurate or unfair links between obesity and health conditions. Many participants felt that the Cancer Council campaign claim (linking obesity with increased risk of cancer) was unreasonable when many thin people also developed cancer:

*"My sister had stage three Cervical Cancer and she's a size 8... cancer can happen to anybody, so I don't think that there's any link there." *(Female, aged 31)

The Cancer Council campaign encouraged individuals to order an obesity prevention kit that included a tape measure to measure their waist and tips for healthy lifestyles. Yet many participants who had ordered the kit found it unhelpful and concluded that the campaign was poorly thought through:

*"I thought, yeah, all right, I'll have that booklet and the tape measure. Well the tape measure wasn't big enough to measure me. Now I am fat, but considering it was aimed at overweight people, you think they might have thought about a tape measure that went around people's waist. So I just thought what a colossal waste of money." *(Male, aged 36)

Others stated that they thought campaigns like the one promoted by the Cancer Council created a 'fat phobic' environment by emotively equating 'thinness' with good health and wellbeing. Some were concerned that this type of campaign would promote eating disorders for individuals of all shapes and sizes. Others stated the assumptions in the Cancer Council campaign discriminated against obese people by automatically reinforcing that "*the definition of obesity automatically equals unhealthy"*. Terms used to describe the campaign included "*degrading*" and "*belittling*". Some stated it made them feel "*depressed*" and *"hopeless"*.

Participants particularly disliked the negative approach of the campaign and the 'scare tactics' used. Some commented that these types of campaigns were excellent examples of the complete lack of understanding of what it is like to be 'fat' in society - and the constant negative reactions to being overweight. Many stated that this approach failed to take into account the complex environmental, social and individual factors that influenced physical activity and food choices:

*"There's a fine line between trying to scare people to death and actually encouraging them." *(Female, aged 36)

*"You can't explain to a six or even seven or eight year old, well Mum's not going to die because the belts not around my waist. My girlfriend's kid sat there and cried because she thought her Mum was going to die because the tape measure wouldn't go around her waist. To my way of thinking scare tactics don't work." *(Female, aged 48)

Rather, they advocated for campaigns based on positive messages and incentives:

*"Targeting people who are overweight is just victim blaming. I don't think that really reaches people who are obese." *(Female, aged 50)

*"I like it when it's more positively directed and I think that can be far more effective in encouraging people." *(Female, aged 33)

Participants also commented that campaigns would never be effective until there was appropriate infrastructure and support to help individuals change their lifestyles:

*"It was just telling you what you should do and a lot of people know that they should be doing that sort of stuff anyway." *(Female, aged 31)

Rather, participants stated that they needed support to uptake the message. Many commented that government money should be directed towards supportive and holistic interventions for individuals, including training for health professionals.

### • Individual based interventions

#### Specialised fitness programs

Over half of participants (81, 57%) thought that tailored fitness programs were effective in supporting obese individuals to address their health and wellbeing. Men in particular thought these programs were effective in encouraging weight loss (24, 67%). Women spoke about the benefits of exercise in a more holistic way, saying that there were many health benefits of being fit and active - regardless of weight or size:

*"I love *[the gym]*. So, for me, it's been very effective. From the point of view of the fact you can go there for three half hours a week, and it will make a difference, even just doing that." *(Female, aged 42)

Participants felt that specialised fitness programs for obese adults and safe spaces (such as women only gyms) could help alleviate the stigma that made obese individuals "*uncomfortable*" and "*self-conscious*" when engaging in activity. Both women and men spoke about the importance of being in an environment where they felt emotionally secure and supported:

*"They help because you feel more secure when you're in a group of women like there's not much judging." *(Female, aged 26)

*"Most overweight people are conscious about how they look in a gym." *(Male, aged 36)

Some stated that current programs were ineffective because they did little to cater for special physical and emotional needs of people who were extremely overweight:

*"Not effective for obese or morbidly obese people because often the equipment can't support their weight." *(Male, aged 55)

Participants felt that this highlighted the urgent need for specialised programs for overweight individuals who wanted to improve their health and wellbeing.

#### Gastric banding surgery

Whilst a third of participants thought that gastric banding surgery was a long term, effective strategy for addressing obesity (44, 31%), most participants doubted the positive claims and marketing of the gastric banding industry and were cautious about its long term success. Some participants stating that the procedure had not been around long enough to make widespread claims about its effectiveness:

*"I haven't heard of any research that is saying that it does help people to keep it off. I don't know whether it has been around long enough." *(Female, aged 53)

Some participants had highly emotive responses to the use of gastric banding surgery. In particular they were concerned about the commercial marketing of the surgery, describing it as the commercial exploitation of an intervention that should only be reserved for individuals in desperate and acute need of medical intervention:

*"Absolutely aghast! Somebody is making a lot of money out of this. It's either drug companies or bariatric surgeons." *(Female, aged 58)

Individuals were also concerned about both the short and long term risks associated with the surgery:

*"I think that's a really bad idea, seriously, seriously bad idea. The things I've been reading about gastric bypass surgery and lap bands and things, it's messing around with a perfectly good digestive system and there are deaths and permanent problems because of it." *(Female, aged 36)

*"To go into surgery where there was a risk that I could die, as an easy fix, sort of didn't sit well with me." *(Female, aged 47)

Others had formed an opinion about the surgery's effectiveness based on the experiences of friends and family members who had had the surgery:

*"I've had a friend who has just gone through it and when she explained the surgery and what went on I'm thinking how you can possibly be healthy after you've had that done." *(Female, aged 32)

*"Don't tell me that weight loss surgery is the way to do it, because I know lots of fat people who have had weight loss surgery and are still fat." *(Female, aged 51)

Participants who thought gastric banding surgery was a positive intervention for obesity were generally those who were waiting to have the surgery. One participant described her own personal short term success with surgery - interestingly describing her weight loss achievement in commercial language:

*"I've lost 17 kilo's now...I'm a walking advertisement for lap banding these days." *(Female, aged 43)

The vast majority of participants were concerned about the mainstream use of this intervention in treating obesity:

*"Medical interventions should be a last resort and lap banding it's been done like changing underpants at the moment." *(Male, aged 55)

Participants were particularly concerned about the use of surgery for more vulnerable groups - such as teenagers and indigenous communities. Again, many of these opinions were based on their contact with others who had had the surgery:

*"A friend of mine's son *[had] *that... He's seventeen and he lost tons of weight... but he now still eats like a teenager and then just vomits it up. Are they mentally equipped, emotionally equipped for what it means to them? It's the same idea of a quick fix now but what does it really do in the long run?" *(Female, aged 39)

*"Teenagers are still growing and you're giving them a medically induced eating disorder, and I don't think that that's a particularly good way to go." *(Female, aged 36)

Some described the surgery as disempowering by attempting to enforce medical ideals of thinness on a diverse population. Others stated that resorting to this type of intervention meant that we had failed to help and support individuals appropriately:

*"I think that the world is going mad, rather than actually looking at the causes... medicine is going mad for plastic surgery." *(Female, aged 49)

*"If that's what we're having to do for most people, then we've failed society. We haven't really done the right thing by everyone." *(Male, aged 50)

Individuals were also concerned about the lack of complete information about the risks and benefits of the surgery. As such, they were cautious about making conclusions about the effectiveness of the surgery. Rather they felt that whilst this information was missing, it should only be considered as a last resort.

#### Commercial dieting

Only 26 participants (18%) thought that commercial dieting was an effective intervention for obese individuals. Participants' perceptions of the effectiveness of commercial diets were strongly influenced by their own experiences, often using themselves as a 'case study' to explain why dieting was ineffective. They characterised the dieting industry as "*greedy", "a scam" *and *"a money making industry"*:

*"As someone who's been there and done it, I know for a fact that it is a major advertising rip off." *(Female, 46 years)

Ironically, many participants still said that they would turn to commercial dieting to help lose weight and improve their health. This was because they had very little other support available to them in trying to change their health and wellbeing.

Only a small number of participants thought that diets were effective for weight loss. Almost all of these individuals were currently on a diet or were a member of a diet club or support group. They tended to describe the effectiveness of the diet in the exact language used in the marketing of the diet: "*it's an investment" *and *"Weight Watchers is not a diet; it's a lifestyle*". It appeared that in some cases commercial diets were perceived to be successful because of the ongoing support that they provided - rather than the effectiveness of the diet itself. Participants often stated that whilst they were aware that the diet was ineffective, at least it provided an environment in which they could obtain help and support from other obese people:

*"*[It] *keeps you in touch with people who are sort of going with you and motivating you and I think this is the point, it's the motivation that you need to keep going." *(Female, aged 74)

Some participants thought that some commercial diets were better than others. In particular, participants trusted *Weight Watchers *more than other commercial diet companies due to their approach being more *"genuine"*, *"sensible" *and health *"promoting" *than those of other diet companies.

Yet most participants distrusted the commercial marketing techniques of the diet industry, and in particular the seductive way in which they *"lured" *individuals back for repeat business. Many called for regulation of the diet industry:

*"*[Diets] *will only work if you stay on them forever. They make more money by people returning over and over again, than their actual successful people." *(Female, aged 53)

*"They're a very short-term, quick fix answer... you put the weight back on and then you go back - that's how those companies make their money, on their repeat offenders." *(Male, aged 48)

Some also described commercial diets as 'unsafe' because dieting enforced unhealthy eating behaviours and led to poor emotional health outcomes for some individuals. Some stated that diets - particularly those which provided pre-prepared meals - were disempowering because they did not create long term behaviour change:

*"That's not empowering to take hold of your own life, that's giving you a whole heap of frozen crap that I wouldn't eat." *(Female, aged 35)

A perceived lack of help and support appeared to encourage participants to turn to commercial dieting even though they seriously questioned their efficacy, safety and motive.

## Discussion

Obese individuals' perceptions of the efficacy of obesity interventions are largely missing from academic research. This study provides a number of new insights into how and why obese individuals support and uptake different types of interventions. This paper also provides important information about interventions at two different levels: 1) Population prevention and 2) Individual care. Both are important arms of an holistic approach to obesity.

Before discussing the results it is important to note the limitations with a study of this kind. The main issue arises with the sample recruited for this study. The sample in this study is highly educated. Over three quarters of the sample had a least a high school education. Whilst there was very little difference in the attitudes and opinions between participants from different education levels, further work with individuals with lower levels of education will enable more rigorous and robust understanding of their attitudes towards obesity interventions. It is also likely that certain types of people are more likely to volunteer their time for detailed qualitative studies. Given the stigma associated with obesity, it is likely that people who feel more comfortable with and capable of speaking about their experiences volunteer their time for these types of studies. This may explain why, for example, more women than men responded to the study, and why the study is more reflective of individuals from highly educated groups.

Participants supported public health interventions which they perceived were non-judgemental, non-stigmatising and empowered individuals to improve their lifestyles rather than focusing on weight loss per se. Participants in this study were less likely to view interventions as effective if they thought they were stigmatising, or blamed and shamed individuals for being overweight. This was particularly true of the Cancer Council campaign which was seen as blaming and shaming. Whilst these types of campaigns may be designed to encourage change, they appear to be disempowering individuals. Research from other stigmatised health conditions also show that these types of campaigns may actually do more harm than good[30-32]. Encouragingly, messages which are positively framed may actually be more influential in changing behaviour[33]. Further investigation should assess how individuals interact with and respond to current obesity media messaging strategies.

Participants used their own personal experiences and their experiences with second hand information (i.e. through the experiences of friends and family members) when judging the effectiveness of interventions. This was particularly true of the commercially influenced interventions (such as surgery and dieting). These commercial interests and motivations were also tied with perceptions of risk - in particular skewing responses about the benefits versus the pitfalls of gastric banding surgery. This has also been shown in other areas of health: consumer perceptions and experiences of the emotional and physical risks associated with interventions may strongly influence their support and uptake of interventions[34]. In contrast, interventions from not for profit agencies - such as government or community advocacy groups - were more trusted than profit driven industries (such as the weight loss and gastric band industries).

Clearly there is a need to tailor interventions so that they are able to reach a range of different individuals. But these interventions must be viewed as part of a comprehensive approach which embraces long term, accessible, affordable and sustainable supports, rather than quick fix responses. Many participants felt that current population based interventions were not backed up by an accessible range of supports at the community level. Furthermore, those supports that were available were not necessarily appropriately tailored to the needs of obese adults. For example, whilst many individuals thought that tailored fitness interventions were one of the most effective types of interventions, few were able to access such interventions, and found that places such as gyms had limited specialised equipment to suit their needs. A better understanding of how to create safe and supportive spaces for obese individuals to engage in physical activity is urgently needed. Consumer involvement in the revision and tailoring of different layers of interventions at both the population and community level will be important in ensuring that needs are met[35].

Overall, it is important to note that none of the interventions had support from more than two-thirds of participants. This was primarily because individuals thought that interventions did not take into account the complexities of the drivers of obesity, and did not understand the lived experiences of the obese adult. This provides important information in developing more sophisticated measures of public perceptions of a range of different interventions for populations and individuals. Future research should attempt to gain such feedback from a wider demographic to better understand how individuals interact with and respond to interventions. This research should go beyond simplistic evaluations of 'weight loss' as the most important outcome measures, towards understanding how we can more holistically encourage health and wellbeing in obese adults. This is essential in appropriately tailoring interventions to meet the various needs of individuals and populations.

## Conclusion

The results of this study provide a unique assessment of the perspectives, attitudes and opinions of obese adults towards six different interventions currently used in Australia to address obesity. Consumer perceptions of the current obesity interventions highlight the need for greater attention to be dedicated to interventions that support and empower individuals to improve their lifestyle. At the individual level, personalised care planning and long term support systems must be developed to assist obese individuals. At the population level, anti-stigma campaigns and regulation should both be explored. The study highlights the complementary perspective that qualitative lay perspectives can add to current understandings of approaches and responses to the obesity epidemic.

## Competing interests

The authors declare that they have no competing interests.

## Authors' contributions

ST was a Chief Investigator on the study. She designed the study, collected and analysed data, wrote the first draft of the paper and lead the critical review and revision of the paper. SL was the study researcher and PhD Student. She contributed to the design of study, collected and analysed data, and contributed to the drafting and critical reviewing of the paper. JH and DC were also Chief Investigators on the study. They contributed to the study design, interpretation of data, and review of the paper. PK was a study Chief Investigator. He contributed to the study design and review of the paper. All authors have approved the final version of the manuscript for publication.

## Pre-publication history

The pre-publication history for this paper can be accessed here:

http://www.biomedcentral.com/1471-2458/10/420/prepub
